# AntiCD30-Conjugated Antibody Plus Standard BEAM as Conditioning Regimen for Autologous Hematopoietic Stem Cell Transplantation in Systemic Anaplastic Large Cell Lymphoma

**DOI:** 10.3390/hematolrep17010003

**Published:** 2025-01-20

**Authors:** Panayotis Kaloyannidis, Basmah Al-Charfli, Biju George, Charbel Khalil, Nour Al-Moghrabi, Samar Mustafa, Dima Ibrahim, Mohammed Alfar, Firuz Ibrahim, Bassam Odeh, Mohammed Daryahya, Philip Shabo

**Affiliations:** 1 Adult Haematology & Cellular Therapy Department, Burjeel Medical City, Abu Dhabi 92510, United Arab Emirates; basmah.alcharfli@burjeelmedicalcity.com (B.A.-C.); biju@cmcvellore.ac.in (B.G.); charbel.khalil@burjeelmedicalcity.com (C.K.); nour.moghrabi@burjeelmedicalcity.com (N.A.-M.); 2 Nursing Department, Burjeel Medical City, Abu Dhabi 92510, United Arab Emirates; samar.mustafa@burjeelmedicalcity.com (S.M.); mohammed.hamid@burjeelmedicalcity.com (M.D.); 3 Infectious Diseases Department, Burjeel Medical City, Abu Dhabi 92510, United Arab Emirates; dima.ibrahim@burjeelmedicalcity.com; 4 Pharmacy Department, Burjeel Medical City, Abu Dhabi 92510, United Arab Emirates; mohammed.alfar@burjeelmedicalcity.com; 5 Nuclear Medicine Department, Burjeel Medical City, Abu Dhabi 92510, United Arab Emirates; firuz.ibrahim@burjeelmedicalcity.com; 6 Hematopathology Department, Burjeel Medical City, Abu Dhabi 92510, United Arab Emirates; bassam.odeh@burjeelmedicalcity.com; 7 Operations Department, Burjeel Medical City, Abu Dhabi 92510, United Arab Emirates; philip.shabo@burjeelmedicalcity.com

**Keywords:** autologous peripheral blood stem cell transplantation, conditioning regimen, non-Hodgkin lymphoma, systemic anaplastic large cell lymphoma, brentuximab vedotin

## Abstract

**Background/objectives:** The outcome of refractory/relapsed systemic Anaplastic Large Cell Lymphoma (R/R-sALCL), especially for anaplastic lymphoma kinase-1 (ALK-1)-negative disease, remains dismal even after autologous hematopoietic stem cell transplantation (AHSCT). The intensification of both salvage and conditioning regimens, without increasing the toxicity, could improve the outcome of AHSCT in R/R-sALCL. **Methods:** Based on the successful experience of the incorporation of antiD20 monoclonal antibodies in the treatment of B-Cell Lymphomas, we designed a salvage and conditioning regimen incorporating the antiCD30-conjugated antibody (Brentuximab Vedotin, BV) to standard chemotherapy regimens, and we describe herein the clinical course of a patient with AKL-ve, R/R-sALCL, who received salvage regimen BV + DHAP, followed by AHSCT with preparative regimen consisted of BV plus standard BEAM. **Results:** The novel regimen was well tolerated, and no severe adverse effects were noticed. The engraftment was prompt and successful. The patient remained in complete metabolic remission for almost 12 months post-transplant. **Conclusions:** The proposed treatment approach, which combines antiCD30-conjugated antibody with standard salvage and conditioning regimens, demonstrated a completely acceptable toxicity with promising efficacy.

## 1. Introduction

Peripheral T-cell Lymphomas represent an heterogenous group of lymphomas and their clinical course is dependent upon histology, immunophenotype, biological markers, molecular mutations, and specific organ involvement. Systemic Anaplastic Large Cell Lymphoma (sALCL), a subtype of Peripheral T-cell Lymphomas (PTCL), is characterized by the strong and uniform expression of the CD30 molecule, which is a 120 kDa transmembrane protein that belongs to the tumour necrosis factor receptor (TNFR) superfamily, and it is divided in two subtypes accordingly to the anaplastic lymphoma kinase-1 (ALK-1) expression [1,2]. The latter is an important predictive factor, not only because patients with ALK+ve/sALCL usually experience fewer relapses after the initial induction remission treatment as compared to those with ALK-ve disease, but also because of the recent advent of agents which inhibit the activity of ALK-1, offering an effective treatment approach even post failure of the initial treatment, thus resulting in better long-term outcomes for patients with the expression of ALK-1 [3,4,5].

The strong expression of CD30 in sALCL renders it an attractive target for immunotherapy. Brentuximab Vedotin (BV) is the first antibody-drug conjugate that combines an anti-CD30 antibody with the drug monomethyl auristatin E (MMAE). After BV internalization via endocytosis, the MMAE molecules are released into the intracellular space and, following binding to tubulin, disrupt the microtubule network within the cell, leading to cell cycle arrest and apoptosis [6].

Traditionally, anthracycline-based regimens comprise the widely used treatment for sALCL; however, the advent of BV has changed the initial treatment approach, since it has been well documented that the incorporation of BV in the initial treatment significantly improved the outcome in either ALK+ve or ALK-ve/sALCL [7,8]. Nevertheless, the outcome of refractory/relapsed sALCL (R/R-sALCL), especially for those patients with ALK-ve/sALCL remains dismal even after autologous haematopoietic stem cell transplantation (AHSCT) [9,10]. The intensification of conditioning regimens without increasing procedure’s toxicity, could potentially improve the AHSCT outcome in R/R-sALCL. We herein describe the clinical course of a patient with R/R-sALCL who shortly after achieving a complete response to a salvage regimen incorporating BV (1.8 mg/kg, max. dose 180 mg on day 1) plus standard DHAP (Dexamethasone 40 mg days 1–4, Cisplatin 100 mg/m^2^ on day 1 and Cytarabine 2 g/m^2^ bid on day 2), he underwent AHSCT with a preparative regimen consisted of BV plus standard BEAM (BV-BEAM).

## 2. Case Report

**Figure 1:**
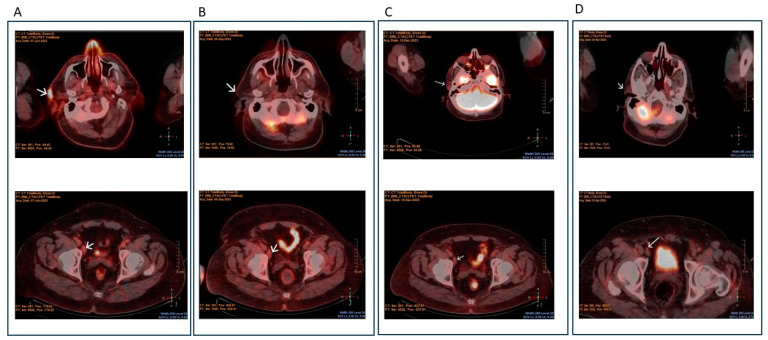
Positron Emission Computed tomography (PET-CT) findings during disease course. (**A**) Before salvage regimen: Hypermetabolic findings due to disease progression are notable in the right pre-auricular area and iliac lymph node (white arrows) (**B**) After salvage regimen with BV+DHAP: complete resolution of the hypermetabolic uptake in the previously notable areas (white arrows). (**C**) Three months after AHSCT with BV + BEAM conditioning regimen: patient considered in sustained complete metabolic remission without detectable disease (white arrows). (**D**) Six months after AHSCT with BV + BEAM conditioning regimen: patient considered in sustained complete metabolic remission without detectable disease (white arrows).

In November 2021, a 55-year-old male with morbid obesity, and no other significant medical or surgical history, was diagnosed with CD30+ve and ALK-ve/sALCL and skin involvement in the right pre-auricular area and generalized lymphadenopathy. He was initially treated in his home country with 6 cycles of standard CHOP, followed by 7 additional monthly cycles of CVP (cyclophosphamide, vincristine, prednisone) as a maintenance therapy. Four months after maintenance completion, he noticed a gradually increased skin lesion in the same anatomical area as at the initial disease presentation (right pre-auricular area), and smaller size lesions in the right leg along with mild oedema, and he visited our department for further evaluation and management. At the initial assessment, the patient was in good general condition, having a noticeable pre-auricular mass of 6 × 5 cm, and few skin lesions in the right leg with mild bilateral lower extremity oedema. The biopsy from the pre-auricular lesion revealed infiltration from large cells, positive for CD30 and for CD3 with CD4 predominance, negative for CD20, ALK-1, EMA, CD56, and Granzyme B, and with a Ki67 mitotic index of 80%, thus confirming progression from the original disease. The Positron Emission Computed tomography (PET-CT) scan, apart from the right preauricular mass, also detected a hypermetabolic iliac node and diffuse fat stranding in the right leg (Figure 1).

Doppler ultrasonography in both lower extremities excluded deep venous thrombosis as the cause of the bilateral oedema. Bone marrow biopsy and aspirate were negative for marrow infiltration from malignant cells. Due to family and social issues, allogeneic stem transplantation (alloSCT) was not feasible, and therefore, the treatment plan was to proceed with salvage therapy followed by AHSCT. Initially, the patient was salvaged with the standard DHAP regimen. Since the marrow was uninvolved by malignant cells, the autologous graft collection was performed after the first salvage and finally a yield of 6 × 10^6^ /kg autologous CD34+ cell was collected. Initially, a good partial response was achieved; however, 20 days later, disease progression was noticed with increased skin lesions in the same pre-auricular area. To control disease aggressiveness, we decided to intensify the 2nd salvage therapy by incorporating the BV to DHAP regimen. Because the patient’s body weight well exceeded 100 kg, he received the maximum total dose of 180 mg of BV, followed 24 h later by DHAP regimen. The regimen was well tolerated, without any unusual or unexpected adverse events. A prompt clinical response was observed, while the PET/CT evaluation confirmed the complete metabolic response of the disease. Since the BV plus DHAP combination resulted in remarkable disease response, we decided to intensify also the conditioning regimen BEAM with the anti-CD30-conjugated antibody. Thus, one day before the initiation of standard BEAM regimen, a dose of 180 mg of BV was administered. The autologous stem cell graft was infused without immediate complications. The engraftment was promptly and successfully performed, and patient achieved ANCs > 500/mm^3^ and PLTs > 50,000/mm^3^ at day +12 and +18, respectively. The early post-transplant period apart of the common side effects (mucositis CTCAE: grade 3, nausea, poor appetite; CTCAE: grade 2), was also complicated with moderate diarrhoea caused by Giardiasis Lambliasis, which was successfully managed with metronidazole and albendazole. Keeping in mind R/R ALK-ve ALCL resistance and aggressiveness even post AHSCT, we decided to continue the BV infusion for 12 more cycles as post-transplant maintenance treatment. The first infusion of maintenance BV was given at day +60, after complete and stable engraftment and after the complete resolution of the toxicities accompanying AHSCT. Currently, the patient is almost 12 months post AHSCT, in good condition, while the primary disease remains in complete metabolic remission (Figure 1). He is tolerating the BV maintenance treatment without experiencing major side effects or myelotoxicity during maintenance treatment; only after the 8th maintenance BV infusion did he complained of a mild and well-controlled peripheral sensory neuropathy (CTCAE: Grade 2).

## 3. Discussion

Despite the recent advances in non-Hodgkin lymphoma (NHL) treatment, the management of ALK-ve/sALCL and especially the R/R disease remains a great challenge [10,11,12]. AlloSCT seems to be more efficacious to control disease refractoriness; however, the high toxicity rates restrict its implementation only in selected patients [12]. On the other hand, AHSCT is accompanied by low toxicity but also by a high probability of disease recurrence [10,13]. In the last three decades, the advent of monoclonal antibodies has dramatically changed the treatment landscape in NHLs. The antiCD20 monoclonal antibody, the 1st immunotherapy used in DLBCL, has been incorporated in all the treatment phases (induction therapy, salvage and conditioning regimens, and maintenance), offering significant better survival outcomes [14]. Later, the CD30-specific antibody-drug conjugate, with a completely different mechanism of action, named Brentuximab Vedotin, offering very promising results, was approved by Food and Drug Administration (FDA) to be incorporated in the treatment of CD30+ lymphomas [15]. However, most of the clinical experience derived from studies related to Hodgkin lymphoma management, and only scant data exist for its use in PTCL management, and particularly for the treatment of R/R-sALCL [7,8,16]. In a phase 2 study where BV was used as single agent in R/Rs-ALCL, the authors reported complete remission (CR) rates of 66%, while for patients consolidated with hematopoietic stem cell transplantation, the 5-year progression free survival estimated almost 70% [17]. In a retrospective study with small series of patients and highly heterogeneous population of PTCLs, the combination of BV with Ifosfamide, Carboplatin, and Etoposide as a first salvage regimen, resulted in overall response rates (ORRs) of 30%, while fewer than 15% of patients achieved CR [18]. A retrospective study from the LYSA group using combination of BV plus Bendamustine in R/R-PTCLs, reported a significantly better ORR of almost 70%, while 50% of patients achieved CR [19]. Nevertheless, none of the reported studies compared the efficacy of BV either as single agent or in combination versus the traditional used salvage regimens. Our patient initially received standard DHAP, and after transient disease control, he experienced disease progression. However, the combination of BV + DHAP resulted in a deep and prolonged remission, being an effective bridge for a successful AHSCT. Since the same patient received the standard DHAP as the first salvage treatment and then combination of BV + DHAP as the second salvage regimen, we can consider our patient the “internal control” for comparing the efficacy of the combination of BV + DHAP vs. the standard DHAP.

To the best of our knowledge, BV has so far not been reported as part of the conditioning regimen prior to AHSCT. The concept of the combination of BV plus BEAM was intended to intensify the efficacy of the conditioning regimen without further increasing the toxicity. The BV+BEAM combination was well tolerated without increasing the overall toxicity rates during the early post-transplant period, while not at all compromising the engraftment process.

One of the commonest reported side effects of BV is severe peripheral neuropathy [7,8]. Our patient did not experience any severe peripheral neuropathy post salvage and shortly after conditioning regimen administration, while even after eight cycles of maintenance with BV, he complained only of mild and completely tolerable peripheral neuropathy (CTCAE: grade2).

In conclusion, the clinical course of our case indicates that the incorporation of BV either in salvage or in preparative regimens prior to AHSCT is feasible and safe and results in promising efficacy. However, its final role in managing RR-sALCL patients eligible for transplant needs to be clarified by prospective studies with a large series of patients.

## Data Availability

All data generated or analysed during this study are included in this article. Further enquiries can be directed to the corresponding author.
